# Starfish (*Asterias rubens*) as a New Source of Marine Lipids: Effect of Season, Size and Oil Extraction Methods

**DOI:** 10.3390/foods11192998

**Published:** 2022-09-27

**Authors:** Ann-Dorit Moltke Sørensen, Adane Tilahun Getachew, Charlotte Jacobsen

**Affiliations:** National Food Institute, Technical University of Denmark, Building 201, Kemitorvet, 2800 Kongens Lyngby, Denmark

**Keywords:** invasive spices, omega-3, phospholipids, supercritical CO_2_, EPA, DHA

## Abstract

The increasing demand for oils that contain health-beneficial omega-3 fatty acids calls for new resources or better utilization of existing resources, such as side-streams or underutilized resources to maintain a sustainable fishery. Starfish has been, until recently, an unexploited resource with limited utilization. Currently, starfish is processed into starfish meal for feed. However, the content of bioactive compounds, such as omega-3 fatty acids and phospholipids, could make it a new source of marine oil containing omega-3 fatty acids for human consumption. The aim of this study was to map the composition of bioactive compounds in starfish and starfish meal at different harvesting times to elucidate the content and variation over seasons. The results showed that starfish is a good source of marine omega-3 fatty acids and rich in phospholipids. Some variation was observed in the composition, especially for EPA bound to phospholipids, which was significantly higher in the spring. Traditional extraction using heat and mechanical separation was not applicable to the starfish, and neither was enzyme-assisted extraction. On the other hand, the supercritical CO_2_ extraction method using EtOH as a co-solvent seemed to be a promising green technology for extracting not only non-polar lipids, but also polar lipids, such as phospholipids. However, the conditions for extraction need further optimization.

## 1. Introduction

Fish oils are rich in healthy long-chain (LC) omega-3 polyunsaturated fatty acids (PUFAs). Due to the health-beneficial effects connected to the consumption of these PUFAs, there is an increasing demand for fish oil for human consumption of approximately 6%/year [1]. In addition, fish oils are also an important constituent of fish feed for farmed fish(aquaculture). Today, fish oil is produced from traditional fish species, such as sand eel, sardines, and anchovies. However, the current supply cannot cover the demand for fish oil for human consumption and aquaculture in a sustainable manner [2]. Therefore, new sustainable raw materials for the production of oils rich in omega-3 PUFAs are needed. Examples of potential new sustainable raw materials for the production of oils rich in omega-3 PUFAs could be underutilized marine biomasses and side-streams from seafood production. Starfish is an example of an underutilized marine biomass, which, until now, has been given limited attention. This is in spite of the fact that the industry considers starfish as a pest, since starfish have no predators and eat mussels, which are otherwise harvested for commercial use [3]. Therefore, the utilization of starfish for the extraction of omega-3 PUFAs will also solve an environmental challenge and benefit the mussel industry. At the moment, the starfish (*Asterias rubens*) is not categorized as an invasive species according to the Ministry of Environment of Denmark [4]. However, the species is considered as invasive, since the population is increasing and has a negative impact on the mussel fishery, and may influence biodiversity as it has no predators. For these reasons, research was carried out in the Western part of Limfjorden (Denmark) in 2013–2015 to evaluate the maximum sustainable yield (MSY) of starfish. It was estimated to be 10,000 tons/year for *Asterias rubens*; however, that number is highly influenced by the mortality and growth of the starfish [3]. In addition, there has been an increasing number of starfish (*Asterias amurensis*) in the North Pacific Ocean in recent years, with a large outbreak in Hokkaido. In this location, large amounts of starfish (approx. 15,000 tons/year) are collected and disposed of as waste [5].

Limited data are available on the composition of starfish. One study has determined the annual variation in the composition of major nutrients in starfish, reporting the fat content to be 30–90 g fat/kg dry matter [6]. Until now, no information has been available on the type of fat, i.e., fractions of triglyceride and phospholipids, pigments, and lipophilic vitamins. However, organs from *Asterias amurensis*, another starfish species observed in Hokkaido (North Pacific Ocean), were shown to be rich in phospholipids, EPA, and DHA [5,7]. The bioavailability of omega-3 PUFAs has been shown to be higher when they are present as phospholipids compared with when they are present as triglycerides [8,9]. According to the composition, there is a great potential for this underutilized raw material to be a source of EPA and DHA with high bioavailability.

Recently, the production of starfish meal for feed applications for pigs and poultry from starfish was started by Danish Marine Protein (DMP, Skive, Denmark; now a subsidiary of Vestjyllands Andel). The extraction of oil from the meal will increase its quality due to the lower risk of lipid oxidation and development of rancidity in the meal.

Therefore, the overall aim of this study was to evaluate the possibility of using starfish as a new sustainable source of marine oils. First, the contents of bioactive compounds in the starfish and starfish meal were characterized with a focus on omega-3 LC PUFAs and if they were present in the form of phospholipids or triglycerides. In addition, the peroxide value (PV) and free fatty acids (FFA) were measured to evaluate the initial quality of the oil. Different sampling points were included to evaluate the variation in the content at different times of the year. Secondly, the possibility of extracting lipids, including omega-3 LC PUFAs, using different extraction methods, ranging from traditional extraction using heat and mechanical separation and enzyme-assisted extraction to the more advanced and environmentally friendly supercritical CO_2_ (Sc-CO_2_) extraction was evaluated.

## 2. Materials and Methods

Starfish (frozen condition) and starfish meal collected from different time points over the year/seasons were received from Danish Marine Protein (DMP; Skive, Denmark; now a subsidiary of Vestjyllands Andel)—see the further description below. Alcalase was provided by Novozymes (Bagsværd, Denmark). All chemicals were of analytical grade and solvents were of HPLC grade. All analyses were performed in duplicate (n = 2).

### 2.1. Raw Material

Starfish were caught in Denmark in the area of Limfjorden (Northern Jytland, Denmark). The starfish were washed after landing (Nykøbing M, Denmark). Catching, landing, and washing were performed on the same day. The washed starfish were stored in containers and transported to DMP (Skive, Denmark).

#### 2.1.1. Starfish

Caught, landed, and washed starfish were sampled in buckets after arrival at the factory and stored in a freezer (−18 °C). The starfish were shipped to the Technical University of Denmark (DTU) frozen and stored at DTU until sorting at −40 °C. The received starfish were thawed, sorted for size, repacked, and re-frozen (−40 °C) until analysis. The starfish were sorted for size based on three different size categories: small < 7.5 cm, medium 7.5–15 cm, and large > 15 cm according to van der Heide et al. (2018) [6]. Table 1 shows the sample codes, sampling points, and sizes of the starfish analyzed.

#### 2.1.2. Starfish Meal

After arriving at DMP, the starfishes were placed in a tank for up to 36 h before chopping. The chopped starfish were pumped into a drying chamber through a closed system for the drying process (drying chamber: 240–280 °C hot air). In the drying chamber, the moisture from the chopped starfish was evaporated and the chopped starfish were transformed into starfish meal (powder) within a few seconds. The dried powder was blown out of the chamber through a bag filter to undergo final sieving before being packed into bags. To improve the stability of the meal, antioxidants, such as tocopherol mixtures, were added during the process. The produced starfish meal was stored in a dry storage room at room temperature before sampling and shipment to DTU. The received starfish meal was stored at −40 °C until analysis. Table 1 shows the sample codes and sampling points for the starfish meals analyzed.

### 2.2. Pretreatment of the Starfish before Analysis

Starfish were slightly thawed and cut into smaller pieces and exposed to liquid nitrogen, after which they were minced using a laboratory blender (Waring, Merck, Darmstadt, Germany) to obtain a fine homogenous mass. This made it possible to extract representative samples for chemical analysis. The pretreated raw material was stored at −40 °C until further analysis.

### 2.3. Dry Matter

Starfish and starfish meal were weighed and placed in an oven overnight (102–105 °C, 20–24 h), after which the dry matter content was determined gravimetrically. The dry matter is expressed as a percentage of the sample weight (*w*/*w*).

### 2.4. Oil Content

Oil was extracted from the pretreated starfish and starfish meal using chloroform and methanol according to the Bligh and Dyer method [10] with a reduced amount of solvent. The oil content was determined gravimetrically after the evaporation of chloroform. The results are reported as a percentage of the sample weight. The obtained lipid extract was used to determine the fatty acid composition, lipid classes, tocopherols, astaxanthin, peroxide value (PV), and free fatty acids (FFAs).

### 2.5. Total Fatty Acid Composition: Fatty Acid Methyl Esters (FAME)

The lipid extracts obtained from the starfish and starfish meal were evaporated under nitrogen. Toluene and heptane with internal standard (C23:0) (1:3 *v*/*v*) were added and the lipids were methylated in a one-step procedure in an acid-catalyzed process with a 20% boron trifluoride reagent. This process was accelerated using a microwave (Multiwave3000 SOLV, Anton Paar, Graz, Austria) with a 64MG5 rotor. The settings for the microwave were 5 min of heating at 500 W followed by 10 min of cooling [11]. After cooling, 1 mL of saturated NaCl solution was added. FAMEs were dissolved in heptane and shaken, and the upper phase (heptane phase) was analyzed on a GC (HP 5890A, Agilent Technology, Palo Alto, CA, USA) according to AOCS Official Method Ce 1b-89 (1998) [12] to determine the composition of the fatty acid methyl esters. For separation, a DB-wax column (10 m × ID 0.1 mm × 0.1 µm film thickness, J&W Scientific, Folsom, CA, USA) was used and the temperature program of the GC oven was as follows: 160–200 °C at 10.6 °C/min, 200 °C maintained for 0.3 min, 200–220 °C at 10.6 °C/min, 220 °C maintained for 1 min, 220–240 °C at 10.6 °C/min, and 240 °C maintained for 3.8 min. The results are reported as the percentages of total fatty acids.

The methylation of the extracted oil obtained after Sc-CO_2_ was carried out in a two-step direct methylation procedure [13], since only a small amount of the fatty acids was converted to fatty acid methyl esters with the one-step methylation method. In brief, 50–100 mg of oil was mixed with 1 mL of NaOH (1 M) in methanol, 1 mL of toluene, and 100 µL of 2% internal standard (C23:0) in heptane and left in an ultrasonic bath (10 min), followed by a boiling step (100 °C, 2 min). The sample was cooled in cold water, after which 2 mL of 20% boron trifluoride in methanol was added and boiled (100 °C, 2 min). After cooling the sample, 2 mL of saturated NaCl solution and 1 mL of heptane with 0.01% BHT were added. The samples were shaken and the upper phase (heptane phase) was analyzed by GC as described for the one-step methylation procedure. 

### 2.6. Lipid Classes 

The separation of lipid classes in the lipid extract was carried out by chromatographic separation on a solid phase consisting of aminopropyl-modified silica (Bond Elute column (Waters, Dublin, Ireland)). A solvent of increasing polarity was used to achieve the desired separation. In the method, the lipids were separated into three groups: neutral lipids (NLs; mainly triacylglycerols (TAGs), but may also include sterol esters, for example), free fatty acids (FFAs), and polar lipids (PLs; including most of the glycerol–phospholipids except for acidic phospholipids (Kim and Salem, 1990)). The following solvents were used for elution: (1) NLs were eluted with a mixture of chloroform and 2-propanol (2:1, *v*/*v*), (2) FFAs were eluted with a mixture of diethyl ether and acetic acid (98:2, *v*/*v*), and (3) PLs were eluted using methanol.

For FAME analysis of the lipid class fractions from the oil extracted with B&D (Section 2.4) from starfish and starfish meal, the fractions (PL and NL + FFA) were evaporated and 1 mL of NaOH (0.5 M) in methanol and 100 µL of 2% internal standard (C23:0) in heptane were added, followed by boiling (100 °C, 5 min). The samples were cooled in cold water, after which 1.5 mL of 20% boron trifluoride in methanol was added, followed by a second boiling (100 °C for 5 min). After cooling the samples, 5 mL of saturated NaCl solution and 2.5 mL of heptane were added, shaken, and the upper phase was used for the FAME analysis. The fatty acid methyl esters were analyzed by GC as described in Section 2.5. 

For the FAME analysis of the lipid classes obtained from the Sc-CO_2_-extracted oil, the NL and FFA fraction was evaporated to near-dryness and the PL fraction was evaporated to 1–2 mL, after which the procedure for methylation was followed as described in Section 2.5 for oil extracted by Sc-CO_2_. 

### 2.7. Tocopherols

Lipid extracts were evaporated to remove chloroform, re-dissolved in heptane, and analyzed for the tocopherol content using HPLC (Agilent 1100 Series, Agilent Technology, Palo Alto, CA, USA) according to AOCS Official Method Ce 8-89 (1997) [14]. Tocopherol homologs were separated using a silica column (Waters (Dublin, Ireland), 150 mm, 4.6 mm, with 3 µm silica film). Tocopherols were quantified by external tocopherol standards using single-point calibration. The results are reported in mg tocopherol/kg starfish or starfish meal.

### 2.8. Astaxanthin, Astaxanthin Esters, and Other Pigments

#### 2.8.1. Astaxanthin and Its Esters 

Lipid extracts were evaporated to dryness under nitrogen, re-dissolved in heptane, injected, and analyzed by HPLC (Agilent Technologies 1100; (Column:Kinetex® 2.6u 100A, 100 × 4.6 mm, Phenomenex) using isocratic elution with heptane:acetone (86:14, *v*/*v*) at a flow rate of 1.2 mL/min. Astaxanthin and astaxanthin esters were detected at 470 nm and quantified against an external standard by using single-point calibration. The results are reported in µg/g of starfish or starfish meal.

#### 2.8.2. Other Pigments Only Measured in One Sample (Starfish Meal December 2019) 

Starfish meal (10 g) and ethanol (70 mL) were mixed, followed by centrifugation (447× *g*, 10 min). The solvent layer was transferred to a tube and filtered (0.22 µm) prior to injection (100 µL) in the HPLC (Agilent Technologies 1100) equipped with an Eclipse XDB-C8 column (4.6 × 12.5 mm, 3.5 µm (Agilent Technologies, Palo Alto, CA, USA)). For the separation on the HPLC, a flow rate of 0.9 mL/min and gradient of Solvent A (70% methanol, 30% 0.028 M tetrabutyl ammonium acetate in water) and Solvent B (methanol) were applied. The following gradient program was applied: 0 min 5% Solvent B, 27 min 95% Solvent B, 34 min 95% Solvent B, 35 min 100% Solvent B, 38 min 100% Solvent B, 40 min 5% Solvent B, and 46 min 5% Solvent B. Pigments were quantified using an external calibration curve with the following pigments: 19-butanal-fucoxanthin, fucoxanthin, lutein, pheophytin a, chlorophyll b, chlorophyll a, and beta-carotene. The results are reported in µg pigment/g sample (starfish meal). 

### 2.9. Peroxide Value (PV)

The PV of the lipid extracts was determined according to the International IDF Standard method [15]. The chloroform in the extract was evaporated and the oil was re-dissolved in a mixture of chloroform and methanol (7:3, *v*/*v*). The PV method applied was a colorimetric method measuring a red-colored complex, ferric–thiocyanate complex, on a spectrophotometer at 500 nm (Shimadzu UV1240, Shimadzu Scientific Instruments, Columbia, MD, USA). The results are reported in meq. peroxides (ROOH) per kg oil.

### 2.10. Free Fatty Acids (FFAs)

The FFAs in the lipid extracts were titrated with NaOH using phenolphthalein as an indicator. The lipid extract (10–15 g) was mixed with ethanol (25 mL) and some drops of the indicator were added. Then, the sample was titrated with 0.1 M NaOH until a faint pink color appeared. The volume of NaOH used for titration was used to calculate the FFA content (%). The results were reported as the amount of FFAs (%) by the oleic acid content of the oil.

### 2.11. Oil Extraction from Starfish and Starfish Meal

To evaluate whether it would be possible to extract starfish oil for human consumption, the extraction of oil from starfish, starfish meal, or both was attempted using different extraction methods. The recovered oil was characterized using B&D extraction, FAME, lipid class analyses, PV, and FFA.

#### 2.11.1. Heat and Centrifugation

Starfish were prepared in two ways prior to oil extraction: (1) Starfish (n = 2) were cut into smaller pieces while still frozen and chopped, and liquid nitrogen was used afterward to obtain a fine homogenous powder using the same method as described in Section 2.2. (2) Starfish (n = 2) were cut into smaller pieces while still frozen and chopped. Before oil extraction, the samples were heated to 90 °C using a water bath and held at 90 °C for 3 min. The oil was mechanically extracted using centrifugation at 12,785× *g* for 25 min (Sorvall RC-6 PLUS, Thermo Fisher Scientific, Osterode, Germany; Rotor: F10s-6x500y). The top layer (oil phase) was recovered after centrifugation.

#### 2.11.2. Enzyme Extraction

Prior to the enzymatic extraction of oil, the starfish were cut into smaller pieces and ground. One sample (two starfish) was ground to a powder with liquid nitrogen, whereas another sample was not. The screening of enzyme-assisted oil extraction was performed with alcalase, which is a commonly used enzyme for oil extraction, and the hydrolysis conditions were within the range of those in other studies [16,17]. Enzymatic extraction was performed using starfish and phosphate buffer (pH 7.4) in a ratio of 1:1 (*w*/*w*) with the addition of 1.5% alcalase (based on the protein content) and carried out for 3 h at 55 °C. After hydrolysis, the enzyme was inactivated by heating the samples to 90 °C for 15 min in a water bath and the samples were centrifuged (40 °C, 15 min, 12,785× *g*). The upper phase was passed through a specialized funnel with a separator to remove water. All phases were stored at −40 °C for further analysis.

#### 2.11.3. Extraction by Heat and Ethanol

Different combinations of heat (60 and 70 °C) and time (10, 20, and 40 min) were applied for the extraction of oil with ethanol (EtOH) from the starfish meal (Dec_2019) to evaluate the effect of time and temperature on the extraction efficiency. The ratio of starfish meal (10 g) and ethanol was 1:7 (*w*/*v*). After extraction, the samples were centrifuged (Sigma 4K15 (Sigma, Osterode am Hertz, Germany) 1400× *g*, 10 min) and the upper phase (100% lipids) was collected, flushed with nitrogen, and stored at −18°C until analysis. The most efficient oil extraction conditions obtained for the starfish meal were then applied to extract oil from the starfish (LA 12 19). The starfish were pre-treated as described in Section 2.2 to a homogenous powder. The starfish were mixed with EtOH before extraction in the same ratio as that used for the starfish meal, and extraction continued in the same manner for the extraction of the oil.

#### 2.11.4. Supercritical Carbon Dioxide (Sc-CO_2_) Extraction with Ethanol

The extraction of starfish meal was conducted using supercritical fluid extraction (SFE) with an MV-10 ASFE System (Waters, Milford, MA, USA). The extraction was conducted with previously optimized extraction conditions [18]. Briefly, 13 g of dried starfish meal (<1 mm) was packed in 25 mL extraction vessels connected to a Sc-CO_2_ inlet and extract outlet line and then stored in the oven. CO_2_ was pumped into the extraction vessel to attain the desired pressure using a high-pressure pump after passing through a cooling heat exchanger. The flow rate of the CO_2_ was 5 mL/min and two different flow rates were set for the co-solvent (EtOH) of 1 mL/min and 3 mL/min. The extraction temperature and pressure were 45 °C and 275 bar, respectively. These parameters were selected based on work reported for the extraction of phospholipids from salmon by-product [18]. The extraction was conducted in one cycle for both static and dynamic extractions. The dynamic extraction stage was conducted under different conditions for a total of 90 min. For the first 70 min of dynamic extraction and 10 min of static extraction, the extraction was conducted in the presence of the co-solvent, and for the last 10 min, the flow of the co-solvent was stopped while keeping the same flow rate of CO_2_ to remove all of the remaining solvents from the extraction vessel. After the extraction was completed, the system was depressurized, and the extract was collected. The remaining solvent in the extract was removed using a stream of nitrogen. Finally, the extract was stored in a freezer at −20 °C until it was required for further analysis. 

### 2.12. Statistics

The results are reported as the average and standard deviation. Multiple sample statistics were performed using Statgraphic (Version 18.1.06, Statpoint Technologies, Inc., Warrenton, VA, USA) followed by Tukey´s post hoc test to identify significant differences between the samples and sampling points. A significance level of α = 0.05 was applied. 

## 3. Results and Discussion

**Figure 1:**
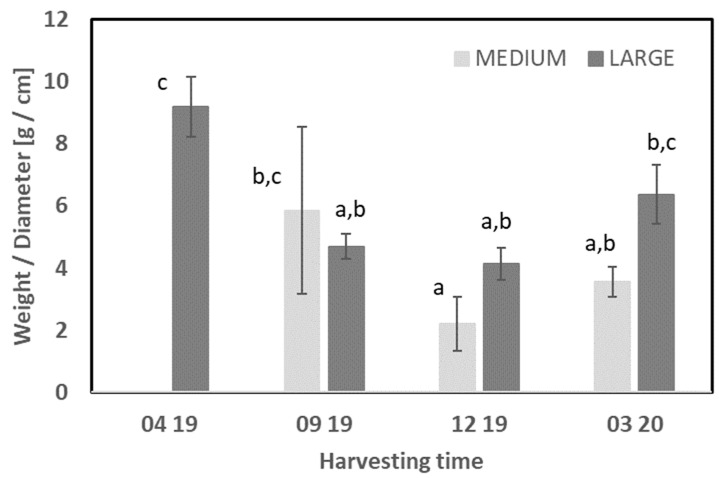
Harvested starfish in biomass per diameter (g/cm). Each column represents an average of three and the error bars indicate the standard deviation. Different letters indicate significant differences. Abbreviation: XX YY indicates the sample month and year, e.g., 04 19 is April 2019.

The weight and size of the starfish were measured and are reported in Table 1, and Figure 1 shows the weight of the starfish by diameter (g/cm). The collected starfish tended to have a higher weight in the spring compared with the autumn. However, the results should be interpreted with caution due to the low sample size. A recent study (2016) evaluated the annual variation in starfish (*Asterias rubens*) depending on the harvest location and size. The results from 2016 showed that the fraction of medium-sized starfish was the largest compared with those of small- and large-sized starfishes in April, June, and December [6]. Another seasonal study on another starfish species, *Asterias amurensis*, collected from the coast of Kushiro city in Japan (Spring—April 2002; Winter—January 2003) showed no significant differences in body weight, and body and arm length; however, the weight of the internal organs was significantly higher in the winter than in the spring, at 92.27 ± 28.57 and 220.9 ± 57.13, respectively [5].

Overall, the results indicate some differences in the weight of biomass/diameter in this study; however, it is not clear if it is a seasonal variation, and more material is needed to draw a further conclusion due to the relatively small sample size in this study. In addition, it could be assumed that the larger weight of biomass could be related to the spawning period of the starfish or be a result of biological variation within the starfishes. 

### 3.1. Compositions and Compositional Variation 

It is known that the type of lipid can affect the extraction efficiency. Therefore, the composition with a focus on lipid amount and type was analyzed before investigating potential approaches for the industrial extraction of lipids for human consumption.

Lipids were separated into three different classes: neutral lipids (NLs), including mono-, di-, and triglycerides), free fatty acids (FFAs), and phospholipids (PLs). Moreover, the contents of other bioactive components, such as tocopherols and astaxanthins, and quality (peroxide value and free fatty acids) were also analyzed. The results obtained are shown in Table 2.

#### 3.1.1. Starfish

The results from starfish show no clear seasonal or size variations in the content of dry matter. For the oil content, a significantly higher oil content was observed in large starfish compared with medium starfish at the different sampling points, and there was a higher oil content in the autumn and winter (LA 09 19 and LA 12 19) (4.0–4.1%) than in the spring (LA 04 19 and LA 03 20) (3.0–3.4%). A recent study reported that the lipid level varied around 40% from 53.0 to 92.4 g/kg of dry matter for starfish harvested from February 2016–January 2017 [6]. These levels of lipid were lower than those observed in the current study (approx. 105–150 g/kg dry matter), which might be the result of the different analytical methods applied for extraction. Van der Heide et al. (2018) [6] hydrolyzed the sample with hydrochloric acid before extraction with petroleum ether for crude fat determination. In addition, starfish in the present study varied less in terms of lipid content, with around 30% variation in the harvest period. The lower variation observed could be partly explained by the different months analyzed. The lowest level of the lipid content in the former study was observed in July and January, which were not analyzed in the current study.

The total content of the important marine omega-3 LC PUFAs (EPA and DHA) ranged from approx. 11 to 21% at the different sampling points. EPA (5.11–15.0%) seemed to vary more between sampling times and starfish sizes compared with DHA (5.16–9.15%). The highest concentration of EPA was observed in large starfish collected in April 2019 and March 2020 (15.0 and 14.6%). These concentrations were significantly higher than those observed for the other sampling points and sizes, except for medium-sized starfish harvested in spring (March 2020).

The content of lipids in the form of phospholipids varied between 21 and 46% of the total lipid content (data not shown). The results from the different lipid classes, phospholipids (PLs), and neutral lipids and free fatty acids (NLs and FFAs), revealed that it was the EPA and DHA, as phospholipids, that mainly varied with significant differences between some sampling points and sizes, whereas, for the fraction of neutral lipids and free fatty acids (NLs and FFAs), no significant differences between sampling points and sizes were observed. As for the total EPA, a significantly higher PL EPA level was observed in the spring for both sizes (medium and large), meaning that the high total EPA level in the spring was at least partly due to a high content of EPA in the membrane lipids (PL). For the PL DHA, a significantly higher content was observed in ME 03 20; however, the difference was not significant from LA 12 19. So far, there are no reports in the literature on the different types of lipids analyzed from this species (*Asterias rubens)* of the whole starfish. However, in earlier studies, organs from another species of starfish, *Asterias amurensis*, were evaluated [5]. Shah et al. (2013) [5] reported high amounts of EPA and DHA in the spring and winter in the polar (phospholipids) and non-polar forms. The contents of PL DHA were 12.7 and 15.5%, non-polar DHA were 8.5 and 14.3%, PL EPA were 28.8 and 36.6%, and non-polar EPA were 20.0 and 8.4% in the spring and winter, respectively. These values corresponded well with the results of the current study on a different starfish species. In contrast, Wang et al. (2013) [7] reported total EPA and DHA levels in *Asterias amurensis* of 19.74% ± 0.44 and 6.60% ± 1.16, respectively. These values are lower than those reported elsewhere for this species, but were within the range of the findings in this study. Thus, the differences observed between our findings and earlier reported findings regarding the EPA and DHA contents could be explained by different species and harvest areas, which could affect the feed types available for the starfish.

Tocopherols are lipophilic antioxidants and can occur as four different homologs, alpha, beta, delta, and gamma. In starfish, only alpha-tocopherol was detected. Significantly higher levels were observed in starfish harvested in September, both for medium- and large-sized starfish, than at other sampling times. For all sampling points, a trend towards a higher tocopherol content in large starfish compared with medium starfish was observed, and for starfish harvested in December, the differences were significant. The large starfish also tended to have a higher content of oil, which could explain the observations for tocopherol (µg/g sample). Astaxanthins, free and in ester form, are lipophilic compounds and naturally occurring carotenoid pigments. This pigment was also detected in starfish. Astaxanthin contributes to a pink color. Besides its colorant property, this compound is also known to have antioxidant properties. For free astaxanthin, the concentrations were low and varied significantly between samples, with concentrations ranging from 0.30–0.77 µg/g. The highest concentration was found in medium-sized starfish from September and December (ME 09 19 and ME 12 19). The concentrations of astaxanthin esters in the samples were higher than those of free astaxanthin and ranged from 1.8–2.8 µg/g with no significant differences between the samples. Bioactive compounds, such as tocopherols, and pigments were not the focus of other earlier starfish studies.

The concentrations of peroxides and free fatty acids (FFAs) can be used as a measure of quality. Lipid hydroperoxides, measured as the peroxide value (PV), are primary oxidation products. For the starfish, the PV level ranged from 5.2–24.9 meq. peroxides/kg oil. A significantly higher PV was observed for the starfish harvested in March 2020, irrespective of size (ME 03 20 and LA 03 20; 3 24.9 and 23.8 meq. peroxides/kg oil). These samples were also among the samples with the lowest concentrations of alpha-tocopherol and astaxanthin. In contrast, starfish with lower PVs also seemed to be among the starfish with higher levels of alpha-tocopherol and astaxanthin. There could be two explanations for this phenomenon. Either the starfish were more prone to lipid oxidation because the feed they had eaten was low in tocopherol and astaxanthin, or the tocopherol and astaxanthin contents were low because they had been degraded when they worked as antioxidants to protect the lipids against oxidation. If the latter was the case, this could indicate substantial oxidation of the starfish during the handling and transportation from the fishing boat to DTU. Nevertheless, all PVs observed in our study were lower than those reported for organs from *Asterias amurensis* (27.7 ± 0.7 meq./kg oil) [7].

FFAs occur as a result of lipase activity. The content of FFAs might be formed from the time of harvest until processing, where heat is applied and enzymes are inactivated. The content of FFA differed significantly and ranged from 3.1–15.7%. The highest level was measured in starfish harvested in December 2019 for the medium-sized starfish followed by the large starfish from the same month.

#### 3.1.2. Starfish Meal

Currently, starfish are dried into starfish meal and used in feed formulations for pigs and poultry. However, the high oil content of the meal and its composition makes it a good source for the extraction of healthy marine omega-3 PUFAs, as seen for starfish. For starfish, some differences were observed between their sizes, i.e., medium and large sizes. All sizes of starfish are used for the production of the meal, and the amount of each size (small, medium, and large) is unknown. The variation in the oil content from B&D extracts from starfish meal was also evaluated in the same sampling period as starfish (Table 2). The dry matter of the meal was 90.6–96.1%, with a significantly higher dry matter content in the later sampling points (September 2019–March 2020, 95.8–96.6%) compared with the two first sampling points (March and April 2019, 90.6%). The oil content varied between 10.7 and 13.1%. EPA and DHA ranged between 5.2 and 8.8% and 3.7 and 5.0%, respectively. The total amount of phospholipids based on total lipids varied from 10% (September) to 20% (March) (data not shown), which was lower than the level in the lipid extracted from the whole starfish. Similar to the findings for starfish, a significantly higher content of EPA was observed in March 2020, but the content of EPA was much lower in starfish meal than in the starfish (8.8% vs. 14.6%). Moreover, the high content of EPA in large starfish in Marchl 2019 was not reflected in a high EPA content in starfish meal from the same month. This could indicate that the meal from this month was prepared from a larger proportion of small starfish with a lower level of EPA. The DHA content in the meal was significantly higher in September 2019 and March 2020 compared with the other sampling points. This could be due to the high content of medium-sized starfish in the meal, as this size of starfish had a significantly higher content of DHA than the other samples.

PL–EPA and PL–DHA varied between 11.1 and 25.4% and 6.4 and 9.0%, respectively. Moreover, the levels of EPA and DHA in the form of triglycerides or free fatty acids were less than half of the level of EPA and half of that of DHA compared with EPA and DHA in the phospholipid form. The level ranged from 3.7–5.2% for NL and FFA–EPA and 3.0–4.5% for NL and FFA–DHA. EPA and DHA in the phospholipid fraction were more different than in the non-polar lipid fraction (NL and FFA) in the starfish meal compared with the whole starfish. A lower amount of PL–EPA was observed in the meal compared with the whole starfish. The highest level of PL–EPA in the starfish meal was observed in March 2020; however, the level of PL–EPA in the meal compared with the concentration in the whole starfish could indicate that the meal was produced from a larger portion of medium starfish than large starfish. In contrast, the level of PL–DHA could indicate the opposite, i.e., the meal was produced from a larger portion of large starfish than medium-sized starfish. Thus, the differences in the observations for EPA and DHA in the phospholipid fraction could either be due to the degradation of the lipids during processing or lower levels being extracted or methylated with the applied methods. The degradation could also explain the higher FFA content observed in the meal compared with the whole starfish. In starfish oil extracted from the meal, up to 40–60% of the EPA was found to be in the phospholipid fraction (March and April), whereas lower levels (23–25%) were found in the other months (September and December 2019). For DHA, the proportion found in the phospholipid fraction was lower (up to 30–33% for spring and autumn and down to 17–19% for winter) (data not shown).

Depending on the production time, different tocopherol homologs were detected. In March and April 2019, both alpha-, gamma-, and delta-tocopherols were detected, whereas in the later periods only alpha-tocopherol was detected. These findings correspond to the observations in starfish. It was assumed that tocopherol was degraded during the production of starfish meal due to the heat used in production. However, different tocopherol blends were added during production to protect the meal from lipid oxidation in the highly sensitive marine PUFAs. This may explain the differences observed between the oil extracted from the different meal production times.

The pigment astaxanthin was also present in the meal; the content of free astaxanthin ranged between 0.3 and 1.0 µg/g meal and that of astaxanthin esters ranged between 2.6 and 4.8 µg/g meal. In addition, the oil extracted by EtOH from the meal also contained other pigments, such as lutein (7 µg/g), pheophytin a (17 µg/g), chlorophyll b (7 µg/g), and chlorophyll a (14 µg/g).

The level of PV was lower in the oil extracted from the meal than in the oil extracted from the starfish and ranged between 0.1 and 12.9 meq. peroxides/kg oil, with the significantly highest level occurring in meal from December 2019, followed by March 2020. Since peroxides are a primary lipid oxidation product and heat is applied during processing, it could be assumed that some peroxides were decomposed into secondary oxidation products. However, this has to be evaluated before further conclusions can be drawn. The level of FFA was between 7.4 and 32.1%, with significantly higher levels in the meals produced in March and April 2019 than in the other meals produced. The reason for the higher FFA content is unknown. It could be assumed to be due to the processing operation; however, it must be evaluated further before conclusions can be drawn.

#### 3.1.3. Summary and Potential as a New Source of Marine Oil

Overall, the compositional results from starfish and starfish meal (Table 2) indicate some variation between the harvested starfish (size and harvest time) and the produced starfish meal. However, these variations do not reflect a clear seasonal variation and could thus be the result of a combination of biological variations, harvest times, starfish sizes, and conditions from harvesting to production. According to Bimbo (1998) [19], quality parameters for crude fish oil are a PV in the range of 3–20 meq./kg oil and a free fatty acid level in the range of 1–7%. A comparison of the evaluated quality of starfish oil (extracted from whole starfish and starfish meal) with the guidelines for crude fish oil showed that the PV was within the range and in the lower half of the guidelines, except for oil extracted from whole starfish from March 2020. However, the FFA% was higher for oil extracted from meal and some starfishes. Both the PV and FFA% can be reduced by a refining process; however, this will also remove pigments and degrade antioxidants. A suggestion for other possible methods to reduce these quality parameters for increased quality may be optimizing the processing of starfish and evaluating the process from harvesting to processing. Wang et al. (2013) [7] concluded from their study on Asterias amurensis organs that simple boiling is a useful method to prevent deterioration (measured by PV and TBA values).

The content of bioactive compounds, especially functional lipids, such as phospholipids and omega-3 LC PUFAs in starfish oil, makes it a high-quality alternative to traditional fish oil produced from sources such as cod liver and anchovies, since these fish oils do not contain phospholipids. Jacobsen (2021) [20] summarized the content of different traditional fish oils, krill, and starfish oils, and showed that starfish oil, similar to krill oil, contained both phospholipids and astaxanthins, but in lower amounts. Thus, marine oil extracted from starfish could be a new source of omega-3 LC PUFAs with different properties to the traditional fish oils.

### 3.2. Extraction of Oil

#### 3.2.1. Traditional Extraction: Heat and Separation

In the traditional industrial extraction of fish oil from sardines, anchovies, and sand eel, heat is applied, followed by a mechanical separation step, pressing, and centrifugation. In this study, we mimicked the industrial process by chopping the starfish into smaller pieces. Additionally, the starfish was also transformed into a fine homogenous powder using liquid nitrogen to increase the surface area even more than chopping and enable the extraction of lipids, followed by heating to 90 °C and holding the temperature for 3 min before centrifugation. Two phases were obtained after centrifugation; however, the upper phase was not a clear lipid phase, indicating inefficient lipid extraction with no differences between the two preparation methods with different surface areas. The lipid content was <0.1% in the upper phase, whereas the precipitate contained 7.1% ± 0.4 lipids. The comparison of lipids in different organs of the starfish *Asterias amurensis* associated with different treatments indicated that boiling the starfish soon after capture facilitated the handling and extraction of the complex lipids, such as the EPA-bound phospholipids compared with untreated (raw) and heated homogenized organs [7]. However, the extraction method used was B&D extraction with chloroform and methanol applied for analytical work, and not the conventional industrial method with heat and mechanical separation for the extraction of fish oil.

A large part of the lipids are present as phospholipids in starfish; hence, it is assumed that this is the reason for limited extraction when only heat and centrifugation were applied. Phospholipids are mainly located in the membrane structure, which reduces the extraction efficiency. As discussed above, the lipid composition in starfish resembles that of krill oil to a greater extent than cod liver oil. Industrially, krill oil rich in phospholipids is extracted from krill meal using a solvent, e.g., EtOH or hexane [21]. However, extraction with solvent does not support green production procedures. Therefore, enzyme-assisted extraction of the LC PUFA from starfish was evaluated using alcalase and heat. Alcalase is an endo-peptidase that cleaves proteins into peptides. Cleaving the proteins in the membrane may be able to release some of the phospholipid-bound EPA and DHA also located in the membrane structure. The attempt to increase the oil extraction yield by enzyme-assisted extraction was unsuccessful. The oil largely still remained in the precipitate after mechanical separation (liquid phase 0.2% and precipitate 6.4% oil). Alcalase has been shown to be able to release lipids from other marine species, including krill [22]. However, their study was carried out with a much higher alcalase concentration than that in the current study based on the protein concentration (approx. 3.5 fold). In the current study, an enzyme concentration of 1.5% based on the protein concentration was used. A higher concentration of enzyme was not evaluated in the current study, as we found that an industrial process would be too costly if a higher concentration of alcalase was necessary.

#### 3.2.2. Combination of Solvent and Heat 

**Figure 2:**
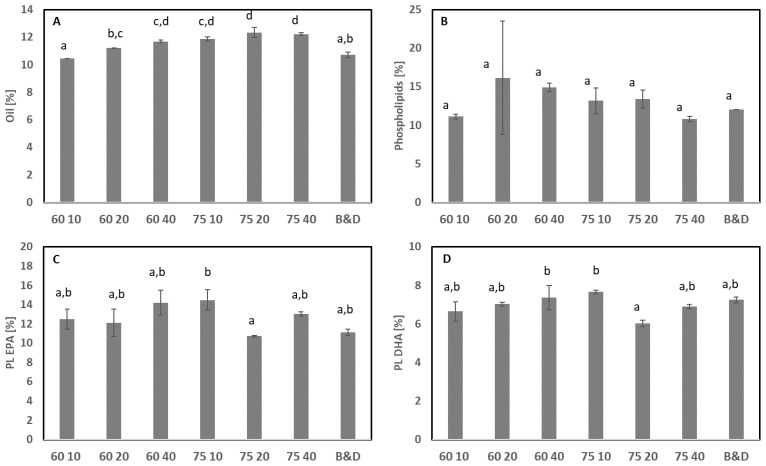
Effect of different extraction conditions (temperature, 60 and 70 °C; time: 10, 20, and 40 min) when lipids were extracted with ethanol. The treatments were compared with B&D extraction. (**A**) Oil extracted (%), (**B**) phospholipids (%) based on oil extracted, (**C**) phospholipid-bound EPA (%), and (**D**) phospholipid-bound DHA (%). Bars and error bars indicate the average (n = 2) and standard deviation. Different superscript letters indicate significant differences. Sample abbreviation: XX YY indicates the temperature (°C) and time (min), e.g., 60 10 is 60 °C for 10 min. Starfish meal from December 2019 was used.

EtOH in combination with heat was also evaluated as an extraction solvent, and we found that it could efficiently extract oil—both non-polar lipids and phospholipids (Figure 2)—from starfish meal. The highest oil level was obtained using 75 °C for 20 and 40 min. However, the oil levels were not significantly higher than those obtained at 75 °C for 10 min and 60 °C for 40 min. The treatment resulting in the lowest oil level was 60 °C for 10 min. This treatment resulted in a significantly lower concentration than the other treatments (Figure 2A). No significant differences were observed in the amounts of total EPA and DHA between treatments. The total amount of EPA was 4.8–5.1% and the total amount of DHA varied less between the treatments, at 4.1–4.3%. The amount of phospholipids extracted varied from 10.8–16.2% of the total lipid extracted. The lowest amount of phospholipids was extracted using B&D, ethanol at 75 °C for 40 min, and 60 °C for 10 min (Figure 2B). The highest amount of phospholipids was extracted at 60 °C for 20 or 40 min followed by extraction at 75 °C for 10 or 20 min. However, no significant differences were observed due to the large variation within the extracted oils. The extraction of phospholipid-bound EPA was highest after treatment at 75 °C for 10 min; however, it was only significantly different from the lowest concentration obtained after extraction at 75 °C for 20 min (Figure 2C). Extraction at 60 °C for 40 min and 75 °C for 10 min resulted in the highest amount of phospholipid-bound DHA, which was only significantly higher than the extraction at 75 °C for 20 min (Figure 2D). Other studies have also investigated the extraction of krill oil using different solvents [23,24]. The results showed that EtOH resulted in higher extraction yield and higher nitrogen content (presence of lipoproteins) than hexane or a mixture of EtOH and hexane (2:1) [20].

Another study on the extraction of oil from krill meal using different solvents showed that using EtOH and isopropanol led to comparatively higher yields and phospholipid contents, but a lower content of minor components than other solvents. Oil extracted with acetone contained more astaxanthin, vitamin A, and sterols, but had the lowest phospholipid content [23]. Thus, the extracted compounds depended on the solvent used. In the current study, only one solvent was used, but slight variations in the experimental conditions (time and temperature) tended to affect the extraction. However, no significant differences were observed within our experimental design (time: 10–40 min; temperature: 60 and 75 °C).

**Figure 3:**
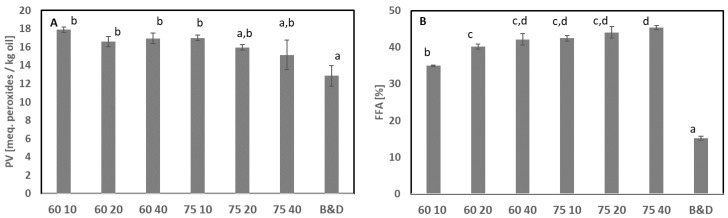
Effect of extraction conditions (temperature, 60 and 70°C; time: 10, 20, and 40 min) on oil quality when ethanol was used as the extraction solvent. The treatments were compared with B&D extraction (B&D). (**A**) Peroxide value (PV) and (**B**) free fatty acids (FFAs). Bars and error bars indicate the average (n = 2) and standard deviation. Different superscript letters indicate significant differences. Sample abbreviation: XX YY indicates temperature (°C) and time (min), e.g., 60 10 is 60 °C for 10 min. Starfish meal from December 2019 was used.

The extraction of oil with EtOH from whole starfish at 75 °C for 10 min resulted in similar results to those obtained from the B&D extraction applied for the characterization (Table 2, LA 12 19). Based on the results from EtOH extraction, it is feasible to extract polar phospholipids with EtOH in combination with heat followed by mechanical separation. For some of the treatments, a significantly higher PV was obtained compared with oil extracted using B&D (Figure 3A). The PV was between 12.9 and 17.9, with a significantly lower PV when using B&D compared with 60 °C at the three different times of extraction and 75 °C for 10 min. In addition, the FFA was also significantly lower in oil extracted using B&D compared with all other oils extracted with EtOH (Figure 3B). Using 60 °C for 10 min resulted in significantly lower FFAs than the other EtOH treatments. The combination of temperature and time increased the FFA, where a higher temperature and longer time resulted in the highest levels. Hence, this method seemed to increase the hydrolysis and oxidation of the lipids. The amount of phospholipids obtained by ethanol extraction was similar to the amount obtained with B&D extraction. This suggests that triglycerides were hydrolyzed to a larger extent than the phospholipids. Therefore, a lower temperature and shorter time would be more favorable in terms of reducing quality deterioration, but lipid extraction was less efficient at 60 °C; thus, other, more efficient extraction methods are required.

#### 3.2.3. Supercritical CO_2_ Extraction without and with Co-Solvent

**Figure 4:**
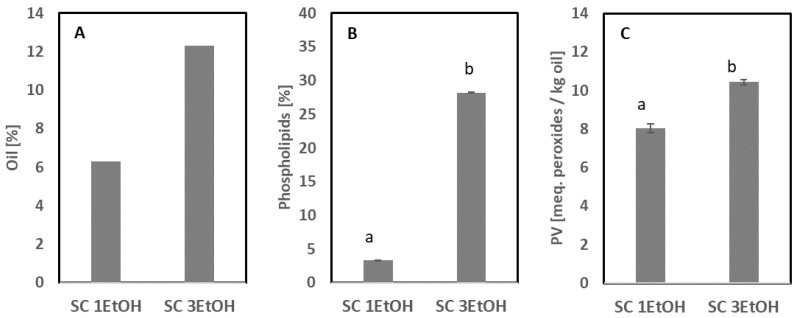
Oil extracted using supercritical CO_2_ extraction with EtOH as a co-solvent at different flow rates (1 and 3 mL/min). (**A**) Oil (%), (**B**) phospholipid (%) fraction after direct methylation with toluene, and (**C**) PV (meq. peroxides/kg oil). Sample codes: SC 1EtOH and SC 3EtOH were extracted with 1 and 3 mL EtOH/min, respectively. Error bars indicate the standard deviation of the analytical measurement of 1 sample (n = 2). Different superscript letters indicate significant differences.

**Figure 5:**
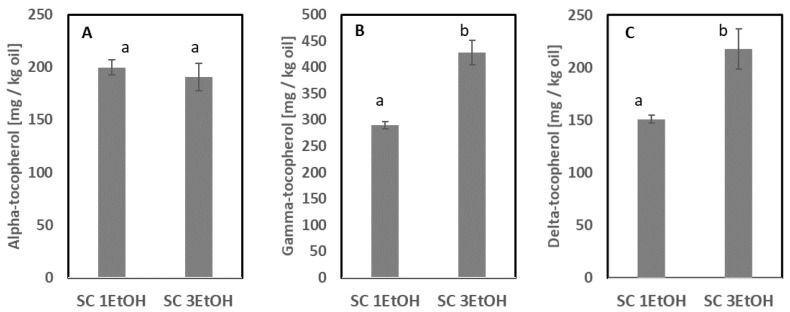
Oil extracted using supercritical CO_2_ extraction with EtOH as a co-solvent at different flow rates (1 and 3 mL/min). (**A**) Alpha-tocopherol, (**B**) gamma-tocopherol, and (**C**) delta-tocopherol (mg/kg oil). Sample codes: SC 1EtOH and SC 3EtOH were extracted with 1 and 3 mL EtOH/min, respectively. Error bars indicate the standard deviation of analytical measurement of 1 sample (n = 2). Different superscript letters indicate significant differences.

Supercritical CO_2_ (Sc-CO_2_) extraction is a more advanced and sustainable extraction method compared with the traditional wet extraction process or extraction with organic solvents, and it can be carried out at a low temperature [25]. Sc-CO_2_ extraction uses CO_2_ as a solvent, which is a readily available, cheap, and environmentally friendly solvent with high selectivity toward lipophilic substances [26,27]. Furthermore, CO_2_ has a relatively low critical pressure (73.8 bar) and is, therefore, an excellent solvent to extract heat-sensitive compounds, such as oils rich in EPA and DHA when found as both neutral lipids and phospholipids [28]. An application trial of Sc-CO_2_ on starfish meal extracted approx. 50% of the oil; however, the phospholipid content was only 0.1% ± 0.04. Thus, the majority of the phospholipids were not extracted in this trial. This is in accordance with earlier reported results on extraction from krill, where the extracted oils were composed solely of nonpolar lipids, mainly triglycerides [29]. It has been reported that the use of EtOH as a co-solvent at lower or higher flow rates with CO_2_ can increase the extraction efficiency of targeted hydrophilic compounds, e.g., phospholipids [30,31,32], which was also confirmed in the current study with EtOH flow rates of 1 and 3 mL/min. The results obtained from Sc-CO_2_ using EtOH at different flow rates as a co-solvent are shown in Figure 4 and Figure 5.

Higher flow rates of EtOH resulted in a higher extraction yield. At a higher flow rate, 12.3% oil was extracted from the meal versus only 6.31% when the lower flow rate was applied (Figure 4A). B&D extraction achieved a yield of 12.1% oil from the meal (Table 2; March 2020). Hence, a similar oil concentration was reached with a high EtOH flow rate using Sc-CO_2_ for extraction. The oil concentrations in the residual meal after Sc-CO_2_ extraction were 1.46% and 6.34% when applying high and low flow rates, respectively. Thus, 90% of the lipids were extracted using an EtOH flow rate of 3 mL/min, whereas only 50% were extracted with a flow rate of 1 mL/min. Not only did the yield increase with the higher flow rate, but also the phospholipid fraction in the oil extracted increased (Figure 4B). With an EtOH flow rate of 1 mL/min, the phospholipid fraction only accounted for 3.3% of the lipids extracted, whereas increasing the flow to 3 mL/min resulted in a phospholipid fraction of 28% of the lipids extracted. B&D extraction from the starfish meal resulted in a phospholipid fraction of 19%, which was lower than that obtained with the high EtOH flow rate.

The PV of the oil extracted from starfish meal by B&D extraction (March 2020, Table 2) was 10.7 ± 0.3 meq. peroxides/kg. The oil extracted using Sc-CO_2_ had a similar level or significantly lower level depending on the EtOH flow rate (Figure 4C). The lowest EtOH flow rate resulted in the lowest PV level.

Additionally, in the oil extracted by B&D extraction from starfish meal (March 2020, Table 2), only alpha-tocopherol was detected, and the other tocopherol homologs were not detected. In the oil extracted by Sc-CO_2_ with EtOH as a co-solvent, gamma- and delta-tocopherols were also present (Figure 5). In the oil extracted with Sc-CO_2_, similar concentrations of alpha-tocopherol were quantified at different EtOH flow rates, whereas significantly higher concentrations of gamma- and delta-tocopherols were quantified in oil extracted with a higher EtOH flow rate. Furthermore, the concentration of astaxanthin ester was not significantly different (1 mL/min: 7.2 ± 1.2 mg/kg oil; 3 mL/min: 13.0 ± 2.3 mg/kg oil). The concentration of free astaxanthin was <0.3 mg/kg oil for both EtOH flow rates, but a significantly higher concentration was obtained with 1 mL/min (0.3 ± 0.01 mg/kg oil).

Sc-CO_2_ extraction with EtOH as a co-solvent thus seems promising as a green technology for the extraction of lipids from starfish. This technique is still in its infancy for processing side-streams from the seafood industry, and it has never been used to extract oil from starfish; however, further optimization with in-depth studies and application trials with the Sc-CO_2_ process are critically required to develop and demonstrate its applicability in industries such as the marine oil industry.

Higher levels of phospholipids (21–46% of the oil) were present in the raw starfish than in the starfish meal (10–20%). This could indicate that the hydrolysis of the phospholipids is occurring in the processing of the meal, and might also explain the slightly higher concentration of FFA in the meal compared with the starfish (Table 2). These results may indicate that the conditions for processing the raw starfish into starfish meal are crucial for the composition and quality of the oil extracted. Therefore, processing parameters, such as the time from harvesting to processing, drying technology, time, and temperature, could be parameters relevant to evaluate for their impact on oil composition and quality.

## 4. Conclusions

The characterization of the starfish for bioactive compounds showed that starfish is a promising source for the production of marine oils rich in omega-3 fatty acids. In addition, starfish can potentially provide benefits other than those of traditional fish oils due to its contents of phospholipids and astaxanthin. Based on the characterization results, differences may occur in the contents of phospholipid-bound EPA in the starfish oil depending on the harvest time, where a significantly higher amount may be present in the spring. This has to be considered in the production of the oil.

All extraction methods are not applicable for oil extraction from the starfish, probably due to its content of phospholipids. The extraction efficiency was very poor using either heat or enzyme-assisted extraction followed by mechanical separation. Sc-CO_2_ extraction using EtOH as a co-solvent resulted in a high extraction efficiency, where phospholipids were also extracted. Thus, this method seems promising as a green technology for this purpose.

There is already an application for starfish meal as a protein source in feed formulations for pigs and poultry. Extracting omega-3 PUFA from starfish meal generates a defatted starfish meal residue. The residue after the extraction of the oil can be used as starfish meal for feed. However, it is assumed that the residue may also be used for other applications with increased value, but this has to be evaluated further before any conclusions can be drawn. Thus, starfish, an underutilized marine species, can be utilized for producing high-quality omega-3 oil to support the increasing demand without the generation of additional side-streams.

## Figures and Tables

**Table 1:** Sample codes for starfish and starfish meal (SM). Starfish sampling (n = 3) month, year, weight, and size (ME: medium; LA: large).

Sample Code	Replicate	Month	Year	Weight (g)	Diameter (cm)
**LA 04 19**	1	April	2019	174	21.5
	2	April	2019	191	20.0
	3	April	2019	198	20.0
**ME 09 19**	1	September	2019	45.9	12.5
	2	September	2019	74.3	8.4
	3	September	2019	67.3	13.4
**LA 09 19**	1	September	2019	89.2	19.3
	2	September	2019	89.7	17.5
	3	September	2019	71.2	16.5
**ME 12 19**	1	December	2019	10.1	8.0
	2	December	2019	31.3	13.0
	3	December	2019	34.0	11.5
**LA 12 19**	1	December	2019	56.8	16.0
	2	December	2019	67.0	15.0
	3	December	2019	79.4	18.0
**ME 03 20**	1	March	2020	38.3	12.0
	2	March	2020	43.6	13.0
	3	March	2020	57.2	14.0
**LA 03 20**	1	March	2020	89.3	16.0
	2	March	2020	122	20.0
	3	March	2020	163	22.0
**SM 03 19**		March	2019		
**SM 04 19**		April	2019		
**SM 09 19**		September	2019		
**SM 12 19**		December	2019		
**SM 03 20**		March	2020		

Grey colored columns indicate that these parameters are not applicable for the meal.

**Table 2:** Characterization of starfish and starfish meal at different sampling times in 2019–2020 (average ± STD, n = 3 starfish and n = 2 starfish meal).

	Dry Matter ^1^	Oil	EPA	DHA	Lipid Classes				Tocopherols ^2^			Astaxanthins ^2^		PV	FFA
					PL EPA	PL DHA	NL + FFA EPA	NL + FFA DHA	Alpha	Gamma	Delta	Free	Esters		
Units	(%)	(%)	(%) of Total Fatty Acids	(%) of Total Fatty Acids	(%) of Total Fatty Acids in the Different Lipid Classes				(µg/g)			(µg/g)		(meq. ROOH/kg oil)	(%) of Fatty Acids
**STARFISH**															
LA 04 19	23.7 ± 1.7 ^a^	3.4 ± 0.1 ^d^	15.0 ± 1.5 ^c^	5.69 ± 0.5 ^a^	28.8 ± 2.6 ^bc^	7.66 ± 1.3 ^a^	6.27 ± 0.4 ^a^	4.60 ± 0.3 ^a^	20.4 ± 3.3 ^b^	ND	ND	0.42 ± 02 ^ab^	1.80 ± 0.1 ^a^	9.47 ± 1.3 ^b^	8.09 ± 0.4 ^c^
ME 09 19	26.8 ± 0.5 ^ab^	3.2 ± 0.1 ^cd^	6.46 ± 0.5 ^ab^	5.37 ± 0.2 ^a^	14.0 ± 1.2 ^a^	8.18 ± 0.3 ^a^	3.30 ± 0.1 ^a^	4.21 ± 0.2 ^a^	30.8 ± 0.4 ^c^	ND	ND	0.77 ± 0.1 ^c^	2.01 ± 0.1 ^a^	7.66 ± 0.3 ^ab^	6.05 ± 0.5 ^b^
LA 09 19	26.6 ± 0.2 ^a^	4.0 ± 0.1 ^e^	7.38 ± 2.1 ^ab^	5.17 ± 0.1 ^a^	15.4 ± 4.0 ^a^	7.76 ± 0.6 ^a^	4.96 ± 2.0 ^a^	4.36 ± 0.2 ^a^	35.4 ± 3.0 ^c^	ND	ND	0.63 ± 0.0 ^bc^	2.38 ± 0.3 ^a^	6.22 ± 0.4 ^a^	5.49 ± 0.0 ^b^
ME 12 19	27.2 ± 1.7 ^ab^	2.9 ± 0.0 ^ab^	6.29 ± 0.6 ^a^	5.38 ± 0.3 ^a^	13.9 ± 2.2 ^a^	9.34 ± 0.5 ^a^	3.48 ± 0.2 ^a^	4.86 ± 0.2 ^a^	8.16 ± 0.3 ^a^	ND	ND	0.71 ± 0.1 ^c^	2.80 ± 0.9 ^a^	9.92 ± 0.8 ^b^	15.7 ± 0.6 ^e^
LA 12 19	31.1 ± 2.7 ^b^	4.1 ± 0.1 ^e^	5.11 ± 0.5 ^a^	5.16 ± 0.0 ^a^	13.9 ± 1.0 ^a^	9.69 ± 0.7 ^ab^	2.47 ± 0.3 ^a^	4.03 ± 0.4 ^a^	17.6 ± 0.7 ^b^	ND	ND	0.55 ± 0.1 ^abc^	2.84 ± 0.3 ^a^	5.22 ± 0.2 ^a^	12.7 ± 0.1 ^d^
ME 03 20	24.5 ± 1.8 ^a^	2.7 ± 0.1 ^a^	11.2 ± 0.7 ^bc^	9.15 ± 0.8 ^b^	24.7 ± 3.6 ^b^	12.6 ± 0.9 ^b^	6.97 ± 2.0 ^a^	9.39 ± 1.2 ^b^	14.2 ± 7.4 ^ab^	ND	ND	0.43 ± 0.0 ^ab^	1.82 ± 0.0 ^a^	24.9 ± 1.6 ^c^	3.09 ± 0.4 ^a^
LA 03 20	25.5 ± 0.6 ^a^	3.0 ± 0.1 ^bc^	14.6 ± 3.6 ^c^	6.59 ± 2.1 ^a^	33.1 ± 3.8 ^c^	8.35 ± 2.1 ^a^	8.38 ± 5.5 ^a^	6.86 ± 2.4 ^ab^	17.8 ± 1.5 ^b^	ND	ND	0.30 ± 0.0 ^a^	2.40 ± 0.3 ^a^	23.8 ± 1.6 ^c^	3.33 ± 0.3 ^a^
**STARFISH MEAL**															
SM 03 19	90.6 ± 0.0 ^a^	11.6 ± 0.4 ^ab^	7.92 ± 0.3 ^b^	3.70 ± 0.0 ^a^	18.0 ± 1.9 ^b^	6.40 ± 0.6 ^a^	5.24 ± 0.2 ^d^	3.16 ± 0.1 ^a^	1.25 ± 0.3 ^a^	18.6 ± 1.6 ^a^	24.0 ± 0.9 ^a^	0.32 ± 0.1 ^ab^	4.76 ± 0.3 ^c^	0.27 ± 0.32 ^a^	32.1 ± 0.5 ^c^
SM 04 19	90.6 ± 0.0 ^a^	11.8 ± 0.0 ^ab^	8.05 ± 0.0 ^b^	3.85 ± 0.0 ^a^	18.8 ± 0.4 ^b^	6.66 ± 0.1 ^a^	5.20 ± 0.1 ^cd^	3.03 ± 0.1 ^a^	1.47 ± 0.1 ^a^	19.0 ± 0.5 ^a^	25.2 ± 0.5 ^a^	0.28 ± 0.1 ^a^	4.44 ± 0.1 ^bc^	0.09 ± 0.1 ^a^	31.9 ± 0.4 ^c^
SM 09 19	96.6 ± 0.1 ^d^	13.1 ± 0.4 ^c^	5.23 ± 0.1 ^a^	4.79 ± 0.1 ^c^	12.1 ± 0.0 ^a^	8.65 ± 0.0 ^b^	4.51 ± 0.1 ^bc^	4.47 ± 0.0 ^b^	24.0 ± 0.3 ^b^	ND	ND	0.58 ± 0.0 ^ab^	3.69 ± 0.3 ^b^	4.89 ± 0.3 ^b^	15.4 ± 0.3 ^b^
SM 12 19	95.8 ± 0.0 ^b^	10.7 ± 0.2 ^a^	5.09 ± 0.0 ^a^	4.32 ± 0.0 ^b^	11.1 ± 0.3 ^a^	7.24 ± 0.1 ^a^	4.41 ± 0.0 ^b^	4.06 ± 0.0 ^b^	22.5 ± 1.0 ^b^	ND	ND	0.62 ± 0.0 ^b^	4.61 ± 0.0 ^c^	12.9 ± 1.1 ^d^	14.8 ± 0.5 ^b^
SM 03 20	96.1 ± 0.0 ^c^	12.1 ± 0.3 ^bc^	8.79 ± 0.2 ^c^	4.96 ± 0.1 ^c^	25.4 ± 0.2 ^c^	8.98 ± 0.0 ^b^	3.71 ± 0.3 ^a^	4.07 ± 0.2 ^b^	265 ± 9.0 ^c^	ND	ND	1.03 ± 0.1 ^c^	2.58 ± 0.1 ^a^	10.7 ± 0.3 ^c^	7.36 ± 0.2 ^a^

^1^ Dry matter for starfish is reported as a percentage of the wet weight, whereas for starfish meal it is reported as a percentage of the meal weight. ^2^ Tocopherols and astaxanthins are given as the concentrations in starfish and starfish meal, i.e., wet weight and meal weight, respectively. LA: Large; ME: Medium; SM: Starfish meal; Sample names followed by two digit numbers (XX and YY) indicate the month and year (see Table 1), e.g., 04 19 is April 2019; EPA: Eicosapentaenoic acid; DHA: Docosahexaenoic acid; PL: Phospholipid; NL: Neutral lipid; FFA: Free fatty acid; PV: Peroxide value; ND: Not detected; Different superscript letters indicate significant differences within the same column and sample type (Starfish or starfish meal).

## Data Availability

Data is contained within the article.

## Abbreviations

DHA	Docosahexaeenoic acid
EPA	Eicosapentanoic acid
EtOH	Ethanol
FAME	Fatty acid methyl ester
FFA	Free fatty acid
LC	Long chain
NL	Neutral lipid
PL	Polar lipid
PUFA	Polyunsaturated fatty acid
PV	Peroxide value
Sc-CO_2_	Super critical CO_2_
